# Stereological analysis of cornu ammonis in prenatally stressed rats: a heuristic neurodevelopmental model of schizophrenia

**Published:** 2014-03

**Authors:** Mohammad Hosseini-sharifabad, Abdoreza Sabahi

**Affiliations:** 1 **Department of Biology and Anatomical Sciences, Shahid Sadoughi University of Medical Sciences, Yazd, Iran**; 2 **Department of Anatomical Sciences, Isfahan University of Medical Sciences, Isfahan, Iran**

**Keywords:** Cornu ammonis, Hippocampus, Prenatal stress, Stereology

## Abstract

***Objective(s):*** The hippocampus has been implicated in pathophysiology of schizophrenia. Prenatal stress is a contributing risk factor for a wide variety of neuropsychiatric diseases including schizophrenia. This study examined long-term effects of prenatal restraint stress on the stereological parameters in the Cornu Ammonis (CA) of adult male rats as an animal model of schizophrenia.

***Materials and Methods:*** Wistar pregnant dams in experimental group were stressed in a cylindrical Plexiglas restrainer daily for 1 hr during last week of gestation. Controls remained in the animal room and were exposed only to normal animal room conditions. At 2 months of age, the volume of the pyramidal cell layer of the CA, the numerical density and the somal volume of the respective neurons were assessed in the male offspring generated from stressed and control pregnancies. Cavalieri's principle, physical disector and nucleator were applied for stereological analyses.

***Results:*** This study showed that prenatal stress significantly decreased the volume of CA3 pyramidal cell layer and the individual somal volume of CA3 pyramidal neurons. However, there were no markedly differences in the numerical density, total number of CA3 pyramidal neurons and stereological parameters in CA1 of prenatally stressed and control animals.

***Conclusion: ***These data indicate that prenatal stress exposure induced neuronal changes in the CA3 subfield of hippocampus which are similar to what is observed in schizophrenia.

## Introduction

Growing evidence suggests that prenatal stress (PS) is a predisposing factor for behavioral and neuroendocrinological changes in later life (1, 2). Prenatal stress represents a well-established experimental protocol that is similar in some respects to schizophrenia, including enhanced response to psychomotor stimulants, prolongation of acute stress-induced secretion of corticosterone, change in sensory gating, deficits in social interactions, and aberrant prefrontal expression of genes involved in synaptic plasticity (3–6).

The hippocampus, an integral component of the corticolimbic circuitry of the brain, which plays a role in the regulation of stress, has interested investigators into schizophrenia for many decades. Prenatal stress can be implicated as a risk factor for developing schizophrenia (7, 8). 

Despite accumulating literature revealing long-term effect of PS on hippocampal function, resembling what is observed in schizophrenia (9–12), only a few studies have examined possible alterations in the developmental anatomy of the hippocampus caused by PS.

Some neuroimaging studies of schizophrenic patients, as well as post-mortem studies, report reduction in hippocampal size in schizophrenia (13, 14). This could reflect a reduction in the size of constituent neurons or reduced number of neurons. However, there are controversial reports concerning size and number change in hippocampal pyramidal neurons in schizophrenia (15, 16). 

Studying the long-term effects of PS in humans is confounded by a high degree of individual variability due in part to the potential confounds such as environmental factors and medications. Animal models, however, can be used to investigate association between prenatal factors and postnatal outcomes, in part because confounding factors can be controlled in the laboratory.

On this background we hypothesized that a restraint stress over the last week of gestation might produce biological results that would be similar to neuronal changes observed in schizophrenic individuals.

Since greater structural abnormalities have been reported in men than in women with schizophrenia (17), we designed a series of experiments to determine the effect of PS on stereological parameters in hippocampal subdivisions of male offspring rats. Consequences of previous studies showed that exposure to PS during the last week of gestation decreased the total volume of hippocampus (18) and caused significant decline in the individual volume of granule neurons in the dentate gyrus without neuronal loss (19); however, other studies indicate a decrease in the number of dentate granule cells in prenatally stressed rats (20–22). 

In continuing our studies on PS, this study aimed to examine the effect of one-week maternal exposure to restraint stress on the volume of somal layers, the total number, and the individual volume of hippocampal pyramidal neurons in the male adult rat.

We employed design-based stereological techniques to determine alterations occurring in the Cornu Ammonis (CA) region (i.e. CA3, CA2 and, CA1) of rat hippocampus due to exposure to PS. 

In this study, the total numbers of neurons in the CA1 and CA3/2 pyramidal cell layers of rat hippocampus were calculated by multiplying the volume of the pyramidal cell layer of each region by the numerical density of the respective neurons. 

Using a set of the vertical sections (23, 24), we applied the nucleator principle (25) for stereological volume estimation of pyramidal perikaria.

## Materials and Methods

***Animals and treatment***

Female Wistar rats (230g – 250 g) were bred in animal house of Isfahan Medical Faculty, and pregnancy was detected by observation of vaginal plugs. The pregnant animals were individually housed with free access to rat feeds (Khorak Daam Pars, Iran) and water in a temperature-controlled (22±2°C) animal room, on a 12 hr light–dark cycle (light on at 07.00–19.00 hr).

The pregnant dams were divided randomly to control and PS groups. Animal care and treatment was approved by Ethical Committee of the Research council of Shahid Sadoughi University of Medical Sciences of Iran.

Pregnant females in the PS group were singly restrained for 1 hr a day (08.00–09.00) in a plexiglas tube (7cm in diameter), from gestational day 15 until delivery. Rats in the control group were not handled except for animal care. After delivery, only 8 to 12 pup, one sex litters were kept for study. On postnatal day 21, the pups were weaned, female and male offspring were separated and housed in groups of four. No more than two male offspring were taken from each litter to reduce ‘litter effects’ (26). The animals were exposed to normal animal room conditions until testing at 2 months of age.

*Histological procedure*

Sixteen rats (n=8 in each group) were deeply anesthetized with urethan (Merk, Germany) and transcardially perfused with a phosphate-buffered solution (pH 7.2, 0.12 mol/l) of 4% formaldehyde and 1% glutaraldehyde. The brains were removed and the cerebral hemispheres were separated by a longitudinal cut in midsagital plane; posterior portion containing hippocampus was collected. One hemisphere was selected at random for estimating both the volume of the CA cell layer and the numerical density of neurons and the other for estimating the volume of individual neurons. 

The brain blocks were processed, at room temperature, as though being prepared for electron microscopic studies. After post fixation in a solution of 2% osmium tetroxide in 0.12 M phosphate buffer, the blocks were rinsed in 25% ethanol and then dehydrated through a graded series of ethanol solutions. After passage through propylene oxide the material was embedded in Epon. Each Epon-embedded block was exhaustively cut into serial, 2 µm semi-thin coronal sections on an ultramicrotome. A total of 2600–2700 sections were cut through the entire hippocampus. From these sections, approximately 13-15 pairs of consecutive sections were systematically and uniformly sampled at random with an initial random start in the first 200 sections, mounted, and stained with toluidine blue for quantitative analysis.

***Estimation of the volumes of pyramidal cell layer of hippocampus***

**Figure 1:**
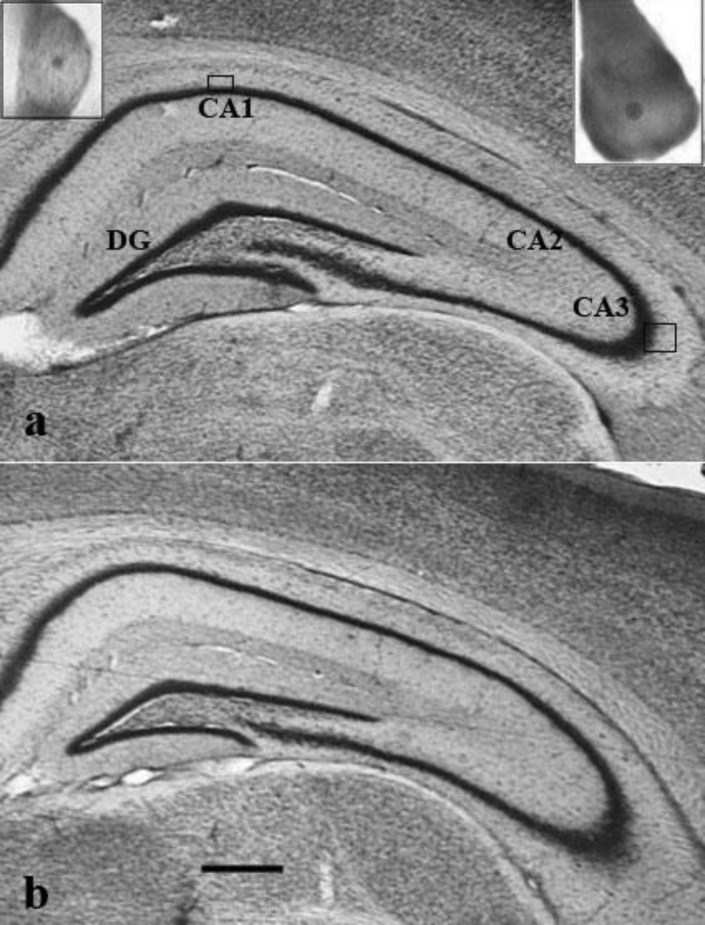
Low-power photographs of the hippocampi of control (a) and prenatally stressed rats (b). Photograph (a) also illustrates neuron containing layers of the hippocamal subdivisions (DG; Dentate gyrus, CA; Cornu Ammonis). Higher magnification (×1800) of CA1 and CA3 pyramidal cells shown in the left and right upper boxes respectively. Scale bar =600 µm and applies to both frames

Discrimination between the different subdivisions of the hippocampal formation was based on cell morphology (27). Cornu Ammonis (CA) was subdivided into CA1, CA2, and CA3 regions (Figure 1). The CA2 region was considered as belonging to the CA3 region because the boundaries between these two fields of the hippocampus are not discrete in conventionally stained sections.

The Cavalieri principle (28) was used to estimate the volumes of the pyramidal cell layers. For this, one section from each consecutive pair was sampled, projected onto a screen at a magnification of ×96 using a Zeiss Microprojector (Zeiss, Germany). 

Each image was superimposed at random, with a grid of systematic uniform test points 10 mm apart. The area of point, a (p), was ≈0.011 mm^2^ in the section plane.

The number of points (P) hitting the layers was multiplied by the area associated with each point, a (p), to obtain an unbiased estimate of sectional area of each profile. The sum of sectional areas of each layer was used to estimate reference volume, V (ref), from the following relationship, where *t* represents the distance between sections; V (ref) = t. P. a (p) 

No area shrinkage correction was used in the study because of the insignificant magnitude of the shrinkage and because no significant difference in shrinkage was found between groups.

***Estimation of the numerical density of neurons***

The numerical density (Nv) was estimated using the physical disector method (29). From the sets of semi-thin sections obtained, photographs of the same area of every region studied were taken from 2 consecutive sections at a final magnification of about 1800. The number (Q^–^) of profiles that appear in one section (‘test section’) but not in an adjacent serial section (‘look-up section’) were counted according to unbiased counting rules using two dimensional unbiased counting frames. 

In this study, because consecutive serial sections were used as the test and look-up sections, the heights (h) of sections were equal to the section thickness (t).

The numerical density (Nv) of neurons in any given region was estimated as: Nv = Q^–^/ah

in which "a" is the total area of the test section examined for that given region.

The total number (N) of each neuronal type is the product of the volume of each specific layer multiplied by the numerical density of a particular cell type in that layer: N= N_V_ × Vref. 

Stereology sampling schemes were chosen for each region so that the sum of the counted neurons would be between 100 and 200 to achieve a sample estimate with a coefficient of error (CE) less than 0.10 (30).

***Estimation of individual neuron volume***

For estimating cell numbers, it is necessary to randomize only the location of section planes. But, for the cell volume estimation, the sections must be cut with a random orientation. Usually, vertical sections (23) are the most efficient for this purpose. We previously described how we made vertical sections from hippocampal slices (19, 23). The bars from each hippocampal slice were embedded in 5% agar. The bars were dehydrated by a graded series of ethanol solutions, 70% (2 5 hr), 96% (2 2 hr), and 99% ethanol (3 2 hr) and infiltrated with glycolmethacrylate (Technovit 7100, Kulzer, Germany) changed daily for 6 days. The bars were embedded at room temperature and the blocks were ready for sectioning after (about) 24 hr.

Each plastic-embedded block was exhaustively cut into serial, 2 µm semi-thin sections with a rotary microtome (MICROM, Germany). The sections were mounted on ordinary glass slides, and immediately dried at 60 C prior to staining.

The sections were stained with a Giemsa stain, briefly; the mounted sections were placed in the staining solution for 70 min at room temperature, rinsed in 1% acetic acid for 3 min, and differentiated in 96% ethanol for 30 min and again in 99% ethanol for 30 min. The sections were dried without coverslips. 

The individual somal volumes of pyramidal cells were estimated using the nucleator (25). Briefly, photographs of the same area of the pyramidal cell layers of the CA1 and CA3 regions were obtained from sets of semi-thin sections of the hippocampus and then analyzed at final magnification of 1800. The pyramidal cells to be measured were sampled in disectors on the vertical sections (measurements of approximately 100 cells usually results in an acceptable variation). Every neuron has the same possibility for being sampled, and they are therefore sampled unbiased in the number distribution. 

The mean volume ($\bar{V}$) of neuronal soma is estimated according to the equation:

$\bar{V}=\frac{4\pi }{3}{\bar{l}}_{n}^{3}$

in which the mean of all cubed lengths (${\bar{l}}_{n}^{3}$) is the distance from the nucleolus of a neuron to the cell boundary. It is estimated with a classifying bidirectional ruler (31). 

To measure the amount of shrinkage caused by fixation and histological procedures, the weight of several tissue bars before processing was converted to a volume through multiplication by the volume/weight ratio (1.04 cm^3^/ g). The volume of each tissue bar after processing and exhaustively cutting was estimated with the principle of Cavalieri and point counting. The volume shrinkage was then calculated as: Volume shrinkage = 1 - (Volume after / Volume before).

Due to the significant magnitude of volume shrinkage (on average 15%) of the glycolmethacrylate embedded sections, the individual somal volumes were corrected for tissue shrinkage.

***Statistical analysis***

Data were then subjected to Independent Samples T Test (Student's t-test). A difference at the level of *P*<0.05 was considered statistically significant. All data represent the mean and the coefficient of variation (CV) where CV= SD/mean.

## Results

**Figure 2:**
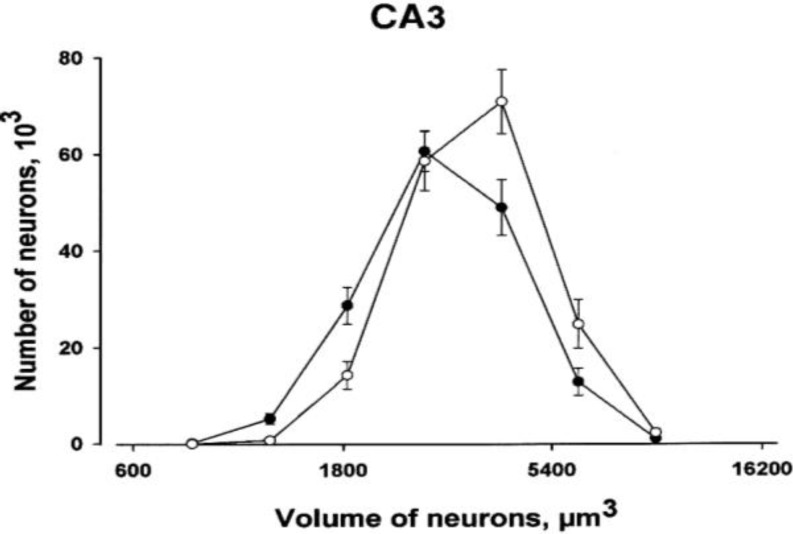
The absolute distribution of unbiased somal volume estimates for CA3 hippocampal neurons. The filled circle indicates the mean distribution of the prenatally stressed (PS) group while the empty circle represents the control group, vertical bars represent SEM The difference in somal volume is easily seen as a left shift of the estimated size distribution in controls compared to PS

Comparisons between the two groups revealed that PS rats had lower volume than controls in the pyramidal cell layers of the hippocampus (Figure 1); however, the reduction in the CA1 pyramidal cell layer was not statistically significant (Table 1). As shown in Table 1, no significant effects of prenatal stress were found on the total number of neurons in CA3 and CA1 hippocampal subfields. Statistical analysis of data revealed significant effect of PS on decreasing the individual volume of the CA3/2 pyramidal cells (Table 1). In PS animals, the somal volume of CA1 pyramidal cells was decreased relative to controls, although this decrease was non-significant (*P*=0.057). The analysis also showed that there was a significant difference in the distribution of somal volume of CA3 pyramidal neurons between the PS rats and controls. Figure 2 shows substantial skewing of the distributions toward smaller somal sizes of neurons in PS rats.

## Discussion

The purpose of the present study was to determine whether restraint stress during the last week of gestation influences the stereological parameters in Cornu Ammonis of hippocampus including the volume of the cellular layers, the total number, and the individual volume of neurons in the pyramidal layers of the offspring. This study was done by applying unbiased stereological methods to achieve highly precise estimates of total number and individual volumes of neurons.

Quantitative results from the stereological analysis showed that exposure to prenatal restraint stress during last week of gestation significantly decreases the volumes of the CA3/2 pyramidal cell layers, where the respective cell bodies were located. Another finding indicates PS decreased the neuronal size in the CA3/2 pyramidal cells of male rat hippocampus. Although, there was a reduction in the mean size of CA1 pyramidal cells, it did not reach the significant level. However, the data of study showed that differences in the total number of neurons in CA3 and CA1 in prenatally stressed rats relative to controls were not significant statistically.

**Table 1:** Comparison of stereological parameters in cornu ammonis (CA) of prenatally stressed (PS) and respective controls

	PS (n=8)	Control (n=8)	*P*
Volume of cell layers (mm^3^)			
CA3/2 pyramidal cell layer	1.90 (0.07)	2.07 (0.08)	0.04
CA1 pyramidal cell layer	1.48 (0.06)	1.56 (0.13)	0.36
Numerical density of Neurons(×10^3^/mm^3^)			
CA3/2 pyramidal cells	84 (0.09)	87 (0.07)	0.36
CA1 pyramidal cells	170 (0.08)	172(0.08)	0.74
Number of neurons (×10^6^)			
CA3/2 pyramidal cells	0.160 (0.14)	0.181 (0.12)	0.07
CA1 pyramidal cells	0.252 (0.10)	0.270 (0.19)	0.41
Individual somal volume (µm^3^)			
CA3/2 pyramidal cells	3037 (0.10)	3588 (0.08)	0.001
CA1 pyramidal cells	1482 (0.13)	1622 (0.09)	0.057

All values presented as mean (CV); The coefficient of variance CV=SD/mean

Based on results, the probable explanation for decreased cellular layer volume in CA3 is reduced neuronal size, rather than loss of neurons. 

Our finding in adult male Wistar rats is in agreement with the results of others (21, 22), where they found no significant reduction in the number of hippocampal granular and pyramidal neurons in male rats (but not female). However, there is conflicting evidence that indicates prenatal exposure to restraint stress for 45 min, three times a day from the last week of gestation results in neuronal loss in the dentate gyrus of the hippocampus in adult male Sprague-Dawley rats. Lemaire *et al* demonstrated that, in prenatally stressed male rats, an alteration of the total granule cell number could be observed only at 3 months of age and that the total granule cell number progressively declines with increasing age after PS. This discrepancy could be related to several possible factors including the rat strain, stress time, the intensity, and duration of the maternal stress, the age of the rats when tested, the hippocampal region, and the methodology employed for counting neurons (20). 

Our measures of average somal volumes yielded useful data that confirm and extend our previous report which showed that PS induces lower spatial learning capabilities along with decreased dendritic branching of CA3 pyramidal cells (32).

The observed decreases in average somal volumes in neuronal populations of prenatally stressed rats provide clues to disturbances in neuronal connectivity, because somal size has been shown to be correlated with dendritic and axonal architecture (33–35).

Change in the perikaryal or nuclear volume has been considered an indicator of alterations in cell metabolism. The nuclear size reduction might cause a more global effect due to the decreased contents of neurotransmitters, intracellular organelles, or the number of synaptic contacts. (36–38).

To the best of our knowledge, there have been no previous attempts for unbiased estimation of individual volume of CA hippocampal neurons after exposure to prenatal stress. 

Ulupinar *et al* (2006) assessed the effect of prenatal stress exposure on the cerebellar morphology (39). They reported that exposure to maternal restraint stress for 6 hr in embryonic day 7 or 14, causes a significant decrease in mean nuclear diameter of both granule and Purkinje cells. In their study the mean profile diameters were used to estimate the true nuclear diameter.

Although the neuron size may be estimated in a number of ways, volume measurement is quite possibly the most informative parameter. Other parameters, including diameter, transsectional area, or circumferential length, depend not only upon size, but also on shape and orientation of neurons. 

The precise estimation of both neuronal number and neuronal volume allowed us to analyze the distribution of volume of neuronal cell bodies. Our results suggest a shift in the distribution toward smaller somal sizes of CA3 hippocampal neurons due to PS. 

The present atrophy of CA hippocampal neurons may be due to exposure to elevated glucocorticoids during prenatal development. Several studies reported that in the last week of pregnancy, rats exposed to restraint stress produced significant elevations in maternal levels of plasma corticosterone (40, 41). Maternal glucocorticoids readily cross the placenta and can interact with any fetal brain region expressing receptors (42). Abundant literature indicates that PS reprograms the hypothalamic-pituitary-adrenal (HPA) axis, resulting in either increased basal secretion or enhanced stress-related secretion of glucocorticoid hormones (1, 2). Our previous work on same animals has demonstrated that the PS paradigm employed here prolongs increased corticosterone levels in response to acute stress. The increase in circulating glucocorticoids could be involved in the cellular mechanism that produces neuronal atrophy in hippocampus (43–45).

Morphometric investigations in which neuronal parameters such as number and size have been determined (16), support the view that there is no loss of hippocampal cells in schizophrenia (38, 46, 47). Additionally, an influential paper reported the presence of a smaller mean size of hippocampal neurons in schizophrenia (38, 48, 49).

Using the results of this study, it can be suggested that restraint stress during the last week of gestation might induce morphometric changes in the hippocampus structure that would be similar to some of the abnormalities observed in schizophrenic patients. This study also indicated that schizophrenia might be a neurodevelopmental disorder of prenatal origin.

## Conclusion

The present study indicated that exposure of pregnant female to stress during the last week of pregnancy leads to a decline in neuronal size in the CA hippocampal field of adult male rats without neuronal loss. The present results may provide a neuroanatomical basis for the understanding of the reported disturbances in hippocampal dependent behavior and learning in prenatally stressed offspring. Interestingly, the results presented here may shed new light on the pathophysiology of schizophrenia.
